# Diagnostic utility of array-based Comparative Genomic Hybridization in a clinical setting

**DOI:** 10.1186/1755-8166-7-S1-P2

**Published:** 2014-01-21

**Authors:** Frenny Sheth, Chaitanya Datar, Koumudi Godbole, Joris Andrieux, Manisha Desai, Bhumi Patel, Jayesh Sheth

**Affiliations:** 1 FRIGE’s Institute of Human Genetics, FRIGE House, Jodhpur Gam Road, Satellite, Ahmedabad, India; 2 Sahyadri Genetics, Unit of Sahyadri Hospitals, Barve Memorial Complex, J.M. Road, Pune, India; 3 Deenanath Mangeshkar Hospital and Research Center, Erandawane, Pune, India; 4 Laboratory of Medical Genetics, Jeanne de Flandre Hospital CHRU de Lille, Lille Cedex, France

## Background

Submicroscopic genomic imbalances are a major cause of congenital and developmental abnormalities including unexplained Developmental Delay (DD), Intellectual Disability (ID), Autism Spectrum Disorders (ASD) and Multiple Congenital Anomalies (MCA). Submicroscopic imbalances are not visible at the resolution level offered by conventional cytogenetics techniques but could potentially be analysed with array based comparative genomic hybridization (aCGH) which offers high resolution scan of the genome. We present the motion backed by clinical evidence for the diagnostic utility of aCGH in a clinical settings for aforementioned cases.

## Material and methods

aCGH analysis was carried out in 37 non-syndromic individuals that clinically presented with either one or a combination of aforementioned phenotypes. Of which, 13 cases had congenital anomalies with or without mental retardation [Group-1]. Remaining 24 cases presented developmental and learning disabilities with seizure [Group-2]. Conventional cytogenetic [GTG-] banding analysis showed apparently normal chromosomal pattern at 550-band resolution. Hence, all 37 cases were further analyzed using Agilent 60k oligonucleotide array-Comparative Genomic Hybridization (aCGH). Sex matched genomic DNA (Promega Corporation, Madison, WI, USA) was used as reference. Relative fluorescence intensity data was analyzed with the aCGH analysis software v3.4 (Agilent Technologies Inc., Santa Clara, CA, USA) by applying Z-score segmentation algorithm with a window size of 10 points to identify chromosome aberrations. Parents in 12 out 16 families were analysed for genomic imbalances using q-PCR to confirm the mode of inheritance.

## Results

Cryptic quantitative genomic alteration was detected in a total of 16 cases that include 31% [4/13] from group-1 and 50% [12/24] from group-2. The chromosomes involved in submicroscopic alterations were 1p, 2q, 5q, 6q, 7q, 8q, 10q, 11q, 12q, 15q, 18q, 19q, 22q, Xp and Xq. Single copy number variation was detected in 14 cases whereas in the remaining two, multiple CNVs were detected. De novo alteration and parental inheritance was confirmed in 4 and 7 cases respectively. One case is currently under investigation and four cases did not provide consent for further investigation.

## Conclusions

aCGH is currently practiced as a front line diagnostic technique for patients with developmental delay and intellectual disability in various countries. This technique is also being applied to understand why patients get developmental disorders as a part of Deciphering Development Delay (DDD) study. Accurate information of etiology aids in genetic counselling and calculating risk for future pregnancy. Our clinical data indicate the application of aCGH in cases with complex phenotypes and apparently normal karyotype.

